# Involving Older People With Frailty or Impairment in the Design Process of Digital Health Technologies to Enable Aging in Place: Scoping Review

**DOI:** 10.2196/37785

**Published:** 2023-01-27

**Authors:** Emilie Kauffeldt Wegener, Jenny M Bergschöld, Carly Whitmore, Marjolein Winters, Lars Kayser

**Affiliations:** 1 Department of Public Health University of Copenhagen Copenhagen Denmark; 2 Department of Health SINTEF Digital Oslo Norway; 3 School of Nursing McMaster University Hamilton, ON Canada; 4 Smart Homes Eindhoven Netherlands

**Keywords:** eHealth, cognitive decline, frail, aging, cocreation, user involvement, mobile phone

## Abstract

**Background:**

With an increase in life expectancy globally, the focus on digital health technologies that can enhance physical and mental health among older people with frailty and impairment has increased. Similarly, research interest in how digital health technology can promote well-being and self-management of health in older age has increased, including an increased focus on methods for designing digital health technologies that meet the various medical, psychological, and social needs of older population. Despite the increased focus, there remains a necessity to further understand the needs of this population group to ensure uptake and to avoid introduction of additional challenges when introducing technologies, for example, because of poor technological design. The scope is limited to digital health technologies meant to enable older people with frailty and impairment to age in place.

**Objective:**

In this study, we aimed to explore how older people with frailty and impairment are involved in various parts of the design processes of digital health technologies and identify gaps or neglected steps in a user-involving design process. This included a focus on recruitment strategies, contributions, and methods used to address the perspectives, needs, and desires of older people with frailty and impairment in the development of digital health technologies.

**Methods:**

A scoping review was conducted in accordance with the PRISMA-ScR (Preferred Reporting Items for Systematic Reviews and Meta-Analyses extension for Scoping Reviews) reporting from February 2021 to April 2021. Literature searches were conducted in PubMed, Scopus, Embase, and IEEE using a search string covering the concepts of health technology, older people, frailty and impairment, user-centered design, and self-management.

**Results:**

In total, 1891 studies were imported for screening from the initial search. A total of 22 studies were included in this review after full-text screening and manual search. Invitation through partners was the most reported recruitment strategy to involve older people with frailty and impairment in the design process of digital health technologies. Furthermore, they were commonly involved in the final evaluation of the development process. Three main gaps identified were the use of outreach approaches to recruit older people with frailty and impairment in the design process of digital health technologies, description of the value of involvement and outcome of the contribution of participants, and knowledge regarding involvement in all parts of the design process.

**Conclusions:**

Although there is literature on methods for involving older people with frailty and impairment in the design of digital health technology, there is little methodological dialogue on the nuances of how different methods for involvement relate to and shape the outcome of the development process.

## Introduction

### Background

According to the data from the World Health Organization (WHO), the global population aged ≥60 years will increase from 12% to 22% between 2015 and 2050 [1]. This change is further challenged by the existing household structures in the European Union (EU) with increasing numbers of older people living alone. One approach to addressing the known challenges associated with this growing population is to support older people to *age in place*, which is defined as “the ability to live in one’s own home and community safely, independently, and comfortably, regardless of age, income, or ability level” [2]. This can be achieved by creating external environments that support social activities within local communities or by introducing ambient and assistive technologies to support everyday life and activities, often referred to as gerontechnology [3-6]. Of particular importance and specific to this review are technologies, often referred to as digital health technologies, that relate to the management of health conditions in older people [7].

### Inappropriate Technological Design

The design of inappropriate technologies can limit uptake and enhance disability and inequity among older people with frailty and impairment [8]. For example, technologies meant to enhance safety and enable independence among older people with cognitive impairment can be disempowering or dehumanizing if designed and used inappropriately [8,9].

The introduction of new technologies that do not address or fully understand the needs of the end user may pose a challenge [10]. For older people, these challenges may be amplified owing to preexisting impairments or frailty. In using the term frailty, we refer to “a state of physiological vulnerability with diminished capacity to manage external stressors,” which can increase the risk of illnesses, falls, disability, and death [11]. The limited uptake of new technologies among older people has been associated with a misalignment in perceptions between those developing the technologies and older end users [12]. This misalignment frequently leads to either limited uptake or outright rejection [13-17]. Therefore, the involvement of older people who are frail or impaired (ie, experiencing physical or mental impairments such as dementia, aphasia, motor dysfunction, ataxia, hearing, or visual loss) in the development of technology is not only necessary in maximizing uptake but also in realizing the intent of technology to mitigate frailty and enable older people to manage challenges of everyday life despite impairments.

### Involving Older People With Frailty and Impairment

The Food and Drug Administration has provided recommendations on patient engagement in the design and conduct of medical device clinical studies, including obtaining input from patients through meetings, home visits, or web-based follow-up and discussing barriers for recruitment with patient advisers [18]. In addition, the International Organization for Standardization provides guidance on how to ensure the design of products and services with the involvement of end users [19]. These guidelines and recommendations for involving end users in the development of health technology support this work and also demonstrate the increased global attention on this important work.

Although it has been widely accepted to involve end users in the design of new technologies and an increased focus on user involvement among certifying bodies is emerging [20,21], there are no standardized requirements or guidelines on how to involve end users [22]. Also, the academic debate on appropriate methods that involve older people who are physically or cognitively impaired or otherwise understood to be *frail* and allow them to express their needs and desires vis-à-vis technology is lagging. Consequently, there is a need for further knowledge about how to involve older people with frailty and impairment in the design of technology to ensure that their needs and desires are addressed and to better understand what they find meaningful to increase the likelihood of technology adoption.

### Digital Health Technologies

In this scoping review, the focus is limited to how older people with frailty and impairment are involved in the design process of digital health technologies. Digital health technologies are defined by the Food and Drug Administration as “the use of computing platforms, connectivity, software, and sensors for health care and related uses” [7]. In this review, we include eHealth and its underlying terms in our understanding of digital health technologies. “eHealth” is defined by WHO as an umbrella term covering the general use of technologies for health care–related processes, including mobile health, the use of different mobile-based solutions, telemedicine, remote clinical services, and telehealth covering both remote and nonremote clinical services [23].

### Aim

The identification and application of purposeful methods for involving older people with frailty and impairment in the innovation and implementation of digital health technology may be a promising means of ensuring that the technology can fulfill its purpose of enabling such older people to age in place with dignity and on their own terms.

Against this background, this review aimed to explore how older people with frailty and impairment are involved in various aspects of design processes of digital health technologies. This was done to identify gaps or neglected steps in a user-involving design process. This included a focus on recruitment strategies, contributions, and methods used to address the perspectives, as well as the needs and desires of older people with frailty and impairment in the development of digital health technologies.

To this end we pursued the following research questions:

1. What are the characteristics of the participants included in the design processes and how are they recruited?
2. Based on the objective of this study, what are the outcomes? What was the technology developed and how did the participants contribute?
3. What kinds of methods and activities have been used to involve older people with frailty and impairment and when were they involved during the development process?

## Methods

### Overview

The scoping review was conducted as part of an EU-funded collaborative project between the EU and Canada called Smart Inclusive Living Environments (SMILE). The SMILE project is working to support aging in place using eHealth solutions with the aim of enabling older people to live an independent and active life, irrespective of frailty and physical or cognitive impairments, using new technologies developed with and for them. Throughout this review, references will be made to “older people” and “health technologies,” for this work, which refers to older people with frailty and impairment and new digital health technologies, respectively.

We conducted a scoping review following the PRISMA-ScR (Preferred Reporting Items for Systematic Reviews and Meta-Analyses extension for Scoping Reviews) to synthesize knowledge; map existing evidence; and identify concepts, theories, sources, and knowledge gaps [24].

### Eligibility Criteria

#### Inclusion Criteria

Studies were eligible for inclusion if they were published in English and in peer-reviewed journals, including conference papers, in any year as identified by our search strings. Eligible studies had to involve >65-year-old people with frailty and impairment. This includes individuals with cognitive decline or deterioration (eg, dementia), cognitive dysfunction (eg, aphasia), neurocognitive impairment, motor dysfunction (eg, stroke and ataxia), and physical and mental frailty or vulnerability. This information could be self-reported in the study. Furthermore, studies were eligible if perspectives, needs, and desires of older people with frailty and impairment were expressed and included in the development process of a digital health technology. Studies had to include a description of the development or design of digital health technologies. This aligned with the need to understand the methods of involvement in the design of digital health technology.

#### Exclusion Criteria

Table 1 provides an overview of the exclusion criteria. Review articles were excluded to avoid redundancy with respect to the original articles included in the review. Case reports, abstracts, and conference proceedings presenting preliminary data were excluded. Thus, full-text articles published with respect to conferences were not excluded.

The scope is limited to the use of digital health technologies meant to enable older people to age in place. Therefore, studies addressing the development of everyday technologies (eg, electrical appliances, technologies for indoor climate regulation, vacuum cleaners, jar openers, and electric curtains) in general products that are not used for specific health issues were excluded.

The following studies were also excluded: effect studies, such as those that only evaluated user experience or implementation (ie, acceptance, feasibility, effectiveness, and efficacy); and studies in which the methods of involvement were not reported or the involvement was not in the context of development of digital health technologies for aging in place.

**Table 1:** Exclusion criteria.

Exclusion	Label	Remarks
Papers that have not been peer-reviewed	Not peer-reviewed article	Also include case reports, conference proceedings, and abstracts.
Full text not available	Full text not available	N/A^a^
Exclude theoretical papers, that is, no involvement of population (eg, research protocols and theoretical papers)	Protocols	Research (protocols) that is planned but not executed.
If data cannot be clearly identified for the age group or subgroup of >65 years	Population not >65	N/A
If data cannot be clearly identified for the group or subgroup of older people with frailty or impairment	Population not frail or impaired	Definition of frail and impaired; individuals with following disorders: cognitive decline (eg, dementia), cognitive dysfunction (eg, aphasia), neurocognitive impairment, motor dysfunction (eg, stroke, ataxia), frailty, vulnerability (not only social vulnerability).
Exclude studies that address the development of everyday technologies such as electrical appliances and technologies for indoor climate regulation and vacuum cleaners. In general, products not used for specific health issues	Everyday technology	This group should be revisited after first round.
Studies in which methods cannot be clearly identified	Does not report methods of involvement	Definition of involvement: end user’s perspectives, needs, and desires are expressed and included in the development process to an extend beyond focus groups and classical participatory design.
Involvement not for the purposes of development	Involvement not for the purposes of development	Definition of development: development or design of new innovative technologies.
Exclude effect studies	Effect studies	Articles that only evaluate user experience or implementation, that is, acceptance, feasibility, effectiveness, efficacy, and so on.

^a^N/A: not applicable.

### Information Sources and Search

Literature searches were performed in PubMed, Scopus, Embase, and IEEE. After the initial screening process, additional identification of relevant studies was performed using following two strategies: (1) identification of relevant studies in the reference lists of the screened studies and (2) input from experts in a workshop, thereby an additional 11 studies were identified; of these, 1 study met the inclusion criteria.

In PubMed, medical subject heading terms were included (search string for PubMed is listed in Multimedia Appendix 1), whereas in other databases (Scopus, Embase, and IEEE) keyword search was conducted (search strings for Scopus, Embase, and IEEE are listed in Multimedia Appendix 1). In total, 3 medical subject heading terms were included in the search string for PubMed: *Telemedicine established in 1993, Cognitive Dysfunction established in 2012, and aged established in 1966*.

Filters for the search strings included full-text availability and English language and excluded case reports, conference proceedings, abstracts, and nonpeer reviewed articles.

### Screening

In total, 2675 studies were obtained using the search strings for PubMed, Scopus, Embase, and IEE; 922 (34.47%) studies were obtained using the search string for PubMed; 1753 (65.53%) was obtained from Scopus and 0 (0) from Embase and IEEE. A total of 29.35% (784/2675) duplicates were removed using Mendeley before importing to the Covidence database [25], a review software tool developed by the Cochrane Community. In Covidence, 1.12% (30/2675) more duplicates were removed based on the title, year, volume, and author. Duplicates were verified by the authors and removed, leaving 69.57% (1861/2675) studies for screening.

**Figure 1:**
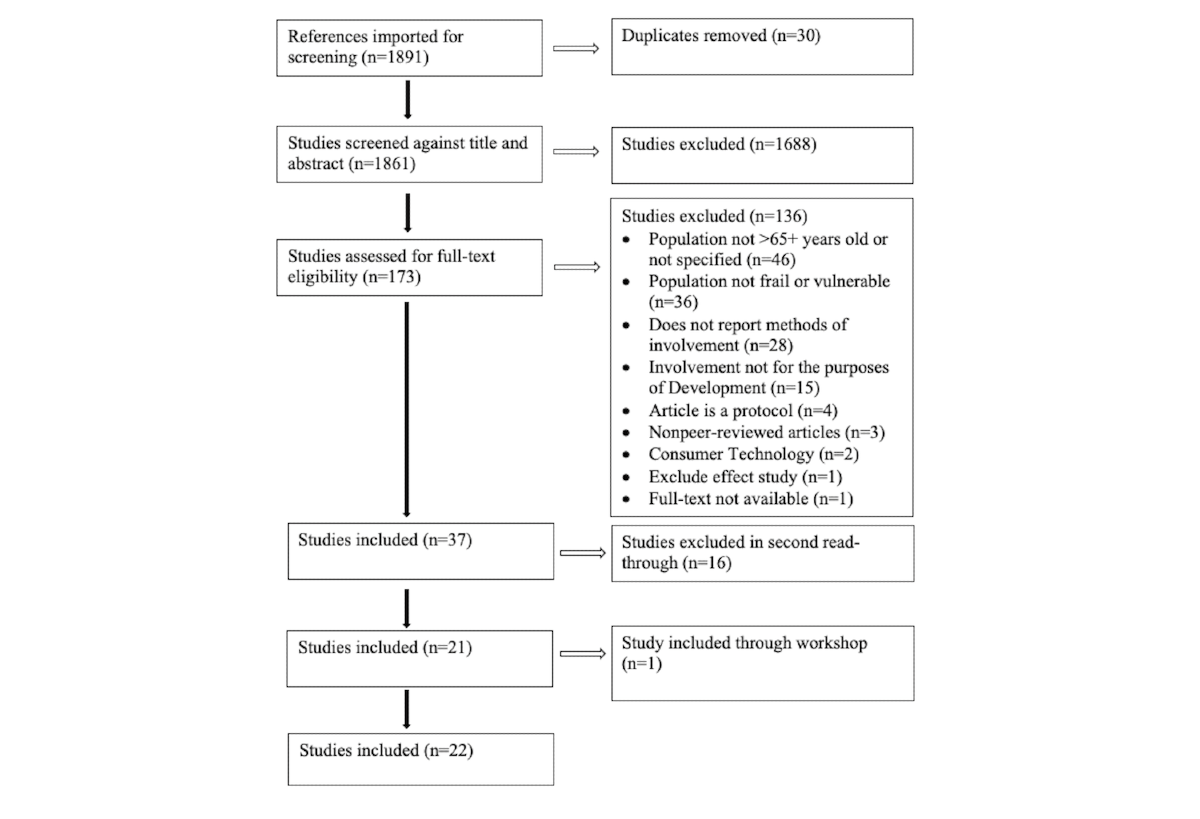
PRISMA-ScR (Preferred Reporting Items for Systematic Reviews and Meta-Analyses extension for Scoping Reviews) reporting flow diagram.

The studies were reviewed using Covidence [25]. After title and abstract screening, 63.1% (1688/2675) studies were excluded, with 10.25% (173/1688) studies then assessed for full-text eligibility. After full-text screening, 2.19% (37/1688) studies were included (Figure 1). During the data extraction phase of the review, 0.95% (16/1688) studies were further excluded because they did not meet the inclusion criteria (eg, only reporting on household appliances or design of future homes or lack of reporting on the involvement of older people in the design process). In addition, 1 study was identified by SMILE project partners in a workshop and was included in the review.

In total, 12.7% (22/173) articles were included. The screening process is illustrated in a PRISMA-ScR flow diagram (Figure 1). The first 4 authors conducted the initial screening by titles and abstracts, and the full-text screening was conducted on a first-to-come basis. All screenings (title, abstract, and full text) involved 4 authors, and each article screening included 2 reviewers. When in doubt about the eligibility of an article, all 5 authors discussed the evaluation. The full-text articles and those retrieved from the manual search and workshop were extracted by the authors EKW, LK, CW, and JMB. All authors participated in the synthesis and presentation of the findings.

Of the 22 studies, 9 (41%) studies had included some population aged <65 years. Because most of the population in these studies was >65 years of age, all the authors decided to include those studies and thus contributes important information about the design of new technologies for the age group of people >65 years.

## Results

### Overview

The following sections present the results of the data extraction. In the first section, a description of the general characteristics of the studies is provided. The second section presents the results related to the characterization of older people with frailty and impairment involved in the design processes and applied recruitment methods. The third section addresses the outcome of the study, the type of digital health technology developed, and contribution of older people with frailty and impairment to the design process. The fourth section addresses the methods and activities used to involve the older people, as well as time point of involvement during the development process.

### General Characteristics of the Included Studies

The 22 studies included in this scoping review were published between 2009 and 2020, indicating that the practice of involving and focusing on how to involve older people with frailty and impairment in the design and development of digital health technologies is an increasing field. The included studies were not limited to a single geographic location. Geographic locations included the WHO Region of the Americas (5 studies from the United States, 1 from Chile, and 1 from Canada), the WHO European Region (2 studies from the United Kingdom; 3 from the Netherlands; and studies from Portugal, Germany, Italy, Finland, and Sweden), the WHO Western Pacific Region (1 study from Malaysia), and the WHO African Region (1 study from South Africa).

Table 2 provides an overview of the population groups included in these studies. Descriptions of the populations show a large representation of somatic conditions, cognitive conditions (eg, dementia and risk of cognitive decline), or a combination of both.

**Table 2:** Overview of conditions represented in studies (N=22).

Author and year	Study population condition	Value, n (%)
Gövercin et al [26], 2010	Risk of falling	1 (5)
Hakobyan et al [27], 2015	AMD^a^	1 (5)
Willard et al [28], 2018	Risk of cognitive decline	1 (5)
Bogza et al [29], 2020	Mild cognitive impairment	1 (5)
Hassan et al [30], 2017 Kerkhof et al [31], 2019	Dementia	2 (9)
de Barros et al [32], 2013 Wannheden and Revenäs [33], 2020	Parkinson disease	2 (9)
Athilingam et al [34], 2017 Grossman et al [35], 2018 Wali et al [36], 2020	Patients with heart failure	3 (14)
Greenhalgh et al [37], 2015 Jacelon et al [38], 2018 Macis et al [39], 2018 Albina and Hernandez [40], 2018	Multimorbidities, known health condition or long-term conditions	4 (18)
Hoffman et al [41], 2019 Alvarez et al [42], 2020 Lehto et al [43], 2013 Oberschmidt et al [44], 2020 Pradhan et al [45], 2020 Vanoh et al [46], 2018 Du Preez and De La Harpe [47], 2019	Different known health conditions or not specified	7 (32)

^a^AMD: age-related macular degeneration.

### Recruitment Strategies

Table S3 (Multimedia Appendix 2) provides an overview of the recruitment strategies used and indicates studies in which no strategy was described. These include purposeful sampling, use of an outreaching approach (eg, attending local community support groups for the relevant study population), and invitation through partners. Moreover, the table provides an overview of the descriptions of the studied populations, including their characteristics and locations.

In 12 (55%) of the 22 studies, recruitment through partners was used to identify relevant and interested older people (for example, through patient associations) [26,28,31-33, 37,38,41,42,44,45,47]. In 4 studies, purposeful sampling was used to recruit participants [35,36,40,46], and in 2 studies, outreach approaches were applied. These included contact made through local support groups for people with age-related macular degeneration (AMD) [27] and through posters and written materials physically placed in local public areas and sent out electronically (ie, through social media) [30]. Finally, in 4 studies, the recruitment strategy was not described [29,34,39,43].

The population groups in the studies were recruited based on specific parameters, including age, health conditions, and living conditions. These parameters can affect the ways in which people can be accessed and recruited. For instance, older people with variations of cognitive decline, such as dementia, are often perceived as difficult to access and are included in the development of new technology. However, in the included studies, older people with dementia and AMD were recruited using different methods, including outreaching (2/22, 9%) [27,30] and through partners (1/22, 5%) [31]. This demonstrates that the otherwise difficult-to-access population groups could be approached and recruited using appropriate approaches, such as an outreaching approach, meaning that a combination of methods of approaching and recruiting participants to target different groups of older people can ensure a broader representation in the design process of digital health technology. However, the most used recruitment method is invitation through partners, in which there is a risk of bias. For example, Oberschmidt et al [44] problematize recruiting through partners and highlight participant bias as a study limitation. The study emphasizes that the older people who participated were very active and outgoing. Thus, it is not representative of all older people. This shows a gap in the knowledge on the use of outreaching approaches to recruit older people with frailty and impairment in the design of digital health technologies.

### Outcome of Involvement

Table S4 (Multimedia Appendix 3) provides a short description of the aims of the studies, as well as an overview of how the included populations contributed to the study outcome. The final column describes the health technologies developed in each study.

In 21 (95%) of the 22 studies, older people lived at home; in 1 (5%) study, the population consisted of inpatients at a hospital [42]. Thus, most of the technologies developed are aimed for people living at home. The technologies developed in these studies include various digital and web-based solutions, such as applications and digital platforms (18/22, 82%) [27-36,38,39,41,42,44-47], assistive technologies (2/22, 9%) [37,40], wearables (1/22, 5%) [26], and interactive caring television (1/22, 5%) [43]. In 64% (14/22) of studies, different variations in outcomes were presented, including those involving participant contributions; for example, how involvement led to a list of themes to be considered when developing an app based on end user needs [36] and how inputs from patients were used to identify design requirements for the interface [35] and to develop a platform [43]. Older people also assessed accessibility, leading to 7 features being included in an apps to prevent delirium in hospitalized older people [42].

Of the 22 studies, 8 (36%) studies did not report or reflect on the participants’ contributions; that is 5 (23%) studies did not report participants’ contributions [28,39-41,47] and 3 (14%) studies described the participants’ contribution in evaluating a prototype or by how they are involved and not by their contribution to the development of the technology [27,31,46]. Thus, gaps in the consistency of description of the value of involvement and outcome of the specific contribution of the participants were identified.

### Involvement Methods Used

Table S5 (Multimedia Appendix 4) provides an overview of the involvement methods used in the studies (eg, surveys and interviews). Moreover, it provides an overview of the time points when the methods were used to involve the participants in the design process, including needs identification, conceptualization, prototyping, or evaluation and further identifies whether the participants were included in one or several parts of the process.

The involvement of older people in this scoping review was assessed based on their involvement in 4 different parts of the development process. These four parts include the following: (1) needs identification, which is the first part of the development process in which end user needs are identified; (2) conceptualization, that is, the conceptualization of the final solution; (3) prototyping, that is, the development of a prototype; and finally, (4) evaluation of the prototype.

In 9% (2/22) of studies, participants were included in all 4 parts of the development process including, needs identification, conceptualization, prototyping, and final evaluation [32,34]. In 23% (5/22) of studies, participants were included in 3 parts of the development process [33,41-43,45], and in 23% (5/22) of studies, older people were included in 2 parts of the development process [27,28,31,35,37]. This overview shows that 12 (55%) of the 22 studies present a combined ecosystem of methods, with consecutive steps that aim to ensure the involvement of older people in different parts of the development process of a digital health technology, from the identification of needs to the generation of ideas, cocreation of a specific product, and final evaluation.

In 45% (10/22) of studies, participants were included in 1 part of the development process. In 60% (6/10) of these studies, the involvement was in the final part of the process, that is, the evaluation of the prototype, using a variety of different involvement methods including, focus groups, workshops, feedback sessions, questionnaire, assessment of acceptance, usability assessment, and rating scales [26,29,30,38,39,46]. Thus, there is a gap in knowledge of the means to involve older people with frailty and impairment in all parts of the design process, including the initial needs assessment phase.

The most frequently used method to involve older people in the included studies (10/22, 45%) was interviews. Interviews were used for needs identification, prototyping, and evaluation. In addition, workshops and focus groups were also commonly reported and applied in all 4 parts of the development process. The participants were mostly involved in the final part of the development process, the evaluation (15/22, 68% of the studies), and in the initial needs identification (13/22, 59% of the studies), whereas participants were least involved in the prototyping process (9/22, 41% of the studies) and conceptualization phase (6/22, 27% of the studies).

Finally, in 14% (3/22) of studies, specific theories were used to inform the analysis [36,44,46]. Du Preez and De La Harpe [47] applied a grounded theory methodology through an iterative and simultaneous process of data collection, coding, category development, and data comparisons to understand the perceptions of older people regarding technologies to support aging in place. Greenhalgh et al [37] position their study within “critical ethnography,” referring to phenomenological philosophy touching upon Maurice Jean Jacques Merleau-Ponty and Martin Heidegger’s work on perception. Finally, Pradhan et al [45] Used a constructivist grounded theory approach in their analysis. In total, 45% (10/22) of studies were conducted based on an existing framework or design concept (eg, feasibility study, scrum, PICTIVE [plastic interface for collaborative technology through video exploration] participatory design, and user-centered design framework) [26,27,31,32,35,36 ,38,39,42,46].

## Discussion

### Overview

This scoping review sought to explore how older people with frailty and impairment are involved in various parts of the design processes of digital health technologies and to identify gaps or neglected steps in a user-involving design process. The focus has been on recruitment strategies, outcomes of involvement, and methods used to involve participants and address their perspectives, needs, and desires.

### Principal Findings

In total, 3 gaps have been identified.

First, a gap in knowledge was identified regarding the use of different outreaching approaches to recruit older people with frailty and impairment in the design of digital health technologies. Involvement does not always begin during the recruitment process. Early involvement will enable an outreaching or alternative recruitment strategy to ensure a broad representation of participants and access hard-to-reach populations. An outreaching approach was effectively used in 2 studies that recruited older people with dementia [30] and AMD [27]. However, the most used recruitment strategy in the current literature is through partners or by purposeful sampling. Second, a gap was identified in relation to the description of the value of involvement and outcome of the specific contribution of the participants. Reflection on and description of the outcomes of participants’ contributions is important. Our findings show that some studies successfully reflected the outcome of participants’ contributions.

In one-third (7/22, 32%) of the studies, the specific outcome of the contribution is not reflected upon, leaving a gap in understanding the degree and value of the involvement process.

Third, a gap was identified in the knowledge regarding the means to involve participants in all parts of the design process, including the initial needs assessment phase. Using a variety of methods to involve older people with frailty and impairment in the design of new technologies is valuable, including focus groups, interviews, and workshops. An identified caution is the underrepresentation of involvement across the full design process as opposed to solely the final evaluation phase.

### Involvement Starts With Recruitment

The findings indicate that choosing the right recruitment strategy is highly important to avoid recruitment bias and initiate a beneficial co-design process for older people with frailty and impairment. Therefore, reflecting on the use of different recruitment strategies is important to access a broader representation.

When recruiting participants, relevant factors should be considered, including how to reach the population of interest, as earlier studies have shown that older people and people with low digital skills are often left out or overlooked in the design process of new technologies. This lack of involvement can lead to increased inequity in health care services and a lack of access to new health technologies for those most in need [48].

The least commonly used recruitment strategy was the outreaching approach. The most used was purposeful sampling and invitation through partners. Oberschmidt et al [44] problematize recruiting through partners and highlight participant bias as a study limitation, emphasizing that the older people who participated were very active and outgoing. Future research need to focus on including older people who are less active and difficult to reach.

Hakobyan et al [27] benefited from using an outreaching approach to recruit people with AMD. The research group established contact with a support group for people with AMD. Over a period of 2 months, the research group attended 4 support group meetings to introduce themselves and learn more about their end users, including their capabilities and limitations. The research group found that the participants reluctance was sometimes related to their participation in research as an experimental subject, rather than an involved *expert* living with their specific condition. Together, the strategy to attend meetings for building relationships and obtaining a deeper insight into the reasons for the hesitation of potential participants ultimately enabled the research group to build a trusted relationship with the support group members, who eventually volunteered to participate in their study. Hassan et al [30] combined posters and written materials and distributed them physically and electronically (ie, via social media and email) to advertise the opportunity for involvement in the study. Using this method, approximately 25 people aged >65 years with dementia, memory problems, and mild cognitive impairments were recruited.

Finally, this scoping review found that some (4/22, 18%) studies that included older people with frailty and impairment had exclusion criteria that might have excluded relevant participants. These include cognitive, visual or hearing impairments, or severely limited dexterity in one or both hands [39], people with dementia [46], and those who required reading skills [38] or at least a secondary level of education (≥7 years) [46].

### Description of Articulated Outcome of Involving Participants

The values of the involvement and contribution of the participants were explicitly addressed in 64% (14/22) studies. However, in 36% (8/22) studies, the contribution of involvement to the outcome was not described. This leaves a gap in the understanding of the degree and value of the involvement process.

The 14 studies addressed user involvement through a description of the involvement or reflection of the involvement. Athilingam et al [34] changed a prototype from being a chest-worn device used to monitor heart rate among patients with heart failure, to being a wrist-worn device, based on input from participants. In the study by Jacelon et al [38] the beta version of the user interface for “ASSISTwell,” a tablet app designed for older people to manage symptoms related to different chronic conditions, was developed using input from end users, retrieved through focus groups. De Barros et al [32] developed 4 apps for smartphone for the self-management of Parkinson disease, including (1) medication; (2) appointments; (3) my day, including disease status and symptoms; and (4) my data, including personal and health information, based on input retrieved through interviews, scoping sessions, focus groups, and usability testing with end users with Parkinson disease. Finally, Grossman et al [35] identified design requirements based on input from end users in the development of an interface to assist older people with heart failure.

In 36% (8/22) studies, the value of involvement and the contribution of the participants were not specified or reflected explicitly. Hakobyan et al [27] aimed through participatory and user-focused research to create a mobile assistive health care–related intervention for people with AMD to promote independent living. The methods used to involve the older people are described, including focus groups, observational studies, and design meetings, but the outcome of the involvement was not addressed. In other studies, participants were involved through surveys [40], interviews, and workshops [47] in the first part of the development process. When older people were involved in the initial and final aspects of design, this was often through interviews and observations [28], in 3 parts of the process including needs identification, prototyping, and evaluation of prototype [41], and in the final part of the design process [39], with no reflection on the outcome of involving the end users.

This could reflect the evolving nature of involving older people in the development of technologies or the lack of description of specific contributions. It could also reflect a lack of actual active involvement and use of specific input from older people; this is not clear in these cases. The number of studies that include older people in the design of technology increased between 2008 and 2020. However, the lack of inclusion of a broader older population, including those with disabilities, remains problematic [12]. Earlier studies found that involving older people in the design of technology has shown beneficial outcomes. These include learning about older people’s needs, adjusting technological designs according to older people’s needs, and a sense of participation among older people. This was emphasized by older participants, who appreciated being part of a generation that used technology [49]. A gap in knowledge about appropriate methods to involve people with disabilities and dementia in technology development remains [50], emphasizing the need for future work focusing on research that includes a broad variety of older people. Future research involving explicit reflections and descriptions could help the development of new ways to involve older people with frailty in the design of new technologies.

### Involvement Methods Used Throughout the Design Process

The findings suggest that different methods, including focus groups, interviews, and workshops, to involve older people in the design of health technologies are valuable. An identified caution from these studies is the lack of involvement in the stages leading up to the final phase of the development process.

In most (15/22, 68%) studies, end users were involved in the final evaluation phase of the development process [26,28-35,38,39,41-43,46]. In 41% (9/22) studies, end users were involved in the third prototyping phase [27,31-34,37,41,42,45], and in 27% (6/24) studies, end users were involved in the second conceptualization phase [32-34,42,43,45]. Finally, 64% (14/22) studies included end users in the first phase “needs identification.” Thus, 36% (8/22) studies did not include end users in the initial “needs identification” of the development process [26,30,31,33,38,39,42,46]. This illustrates an overrepresentation of involvement in the final part of the design process conceptualization and evaluation, where a mix of focus groups, questionnaires, usability assessments, and observations are used. This is problematic considering the need for end user involvement to guide the initial development. Earlier studies suggest that involving end users, including people with dementia, can provide a better understanding of end users’ needs for a better design outcome and have a positive impact on future user experience [50]. Although there is no evidence in the studies stating that involvement in the beginning or the middle of a design process is especially rewarding, this review identifies a lack of involvement in the earlier and middle parts of design processes where needs and desires are normally identified before initiating the conceptualization and prototyping process.

In only 9% (2/22) of studies, participants were included in all 4 stages of the design process. Athilingam et al [34] involved participants with heart failure in initial needs identification through needs assessment interviews. Moreover, the participants answered a questionnaire and provided input to the conceptualization and feedback on the design, features, and ease of use during the development phase. Finally, a feasibility study was conducted leading to significant changes in the software and design, which changed from a chest-worn device to a wrist-worn device. These elements were all a part of the study that focused on “patient engagement” with the purpose of achieving both a well-targeted solution for this specific population group and achieving persistent self-care and self-management, including positive health behavior for this group. De Barros et al [32] included participants through interviews and a scoping session with focus on daily routines, motivation mechanisms, medication-related behaviors, and specific requests for the smartphone app. Furthermore, focus groups and usability testing were conducted throughout the development of a smartphone app for self-management in people with Parkinson disease.

Among the identified methods of involvement, there was no indication that some were more successful than the others in identifying older people’s needs. As the purpose of this review was not to judge how and which kind of involvement method have been beneficial for the outcome of the studies, the findings highlight the methods that may be used to involve older people in design processes, so that their needs and desires are heard. Newell et al [51] stated that classic standards and guidelines for user-centered design are not always appropriate for including older people and people with disabilities. This suggests that “user-sensitive inclusive design” is a new way of including older people. This includes forming a close bond with the participants and using experimental techniques to involve older adults; for example, through theatrical techniques using actors instead of personas to impersonate a diverse group of older disabled adults.

There was no general difference in how and when participants with different conditions are involved. People with cognitive impairment were involved through focus groups, interviews, and workshops and were involved in all parts of the process in various studies. This could indicate that specific diseases are not limited to one specific involvement method and that there are several possibilities in relation to the involvement of older people with frailty and impairment in development processes. However, as several studies failed to report the outcome of involvement, it is impossible to draw strong conclusions about the appropriateness of the specific methods used. Earlier studies address the need for improving traditional methods used to involve end users and to consider limitations related to an aging population, including activities such as interviews, questionnaires, and observations. Moreover, earlier findings suggest that involvement and engagement in the initial steps of a development process increase the potential to create a technology that considers relevant limitations and characteristics related to older people [52].

For future work, consideration of the outcome in relation to the degree of involvement is relevant for further assessment. This could include considering the level of involvement in relation to how many steps of the design process the end users are involved in and defining and assessing the degree of “active involvement” in using the different involvement methods. Further research on the effectiveness of these methods is required.

Recommendations from important stakeholders for engaging end users in the development of new medical and health technologies [20] have not been addressed in any of the included studies. There may be a need for specifications regarding how and in which steps user involvement and engagement should be performed. Our findings do not indicate a reason for excluding older people with frailty and impairment. It may be necessary for the regulatory bodies to clarify that these groups, if relevant to include, should not be excluded.

### Limitations

Publications regarding digital health technology development are often conducted as part of the preparation for a certification process that is required by a funding body or in relation to a specific research goal. We may therefore have missed documentation in relation to commercial product development.

There may also be limitations in the representation of the specific disease addressed. The focus of this study was on older people with frailty and impairment, and thus, an overrepresentation of people with cognitive impairments is present, leaving out other major global disease burdens such as ischemic heart disease, diabetes, or multimorbidity, which may contribute to frailty.

Another limitation can be found in the definition of the applied technology used in this study. In this scoping review, studies involving welfare and mundane everyday technologies with no health-specific purpose were excluded, such as robot toilets, electrical curtains, robot vacuums, robot toilets, automated baths, and so on, as they are also part of a smart living environment and do not necessarily represent specific health technologies that were assessed in this study. Nevertheless, these studies may report the relevant methods on how to involve this population group for technology development, and the limitation to scope of the review is that we did not include those studies per the exclusion criteria.

### Conclusions

This scoping review presents existing knowledge on how older people with frailty and impairment are involved in the design of digital health technologies that can contribute to their aging in place and also identifies gaps or neglected steps in a user-involving design process.

A gap in knowledge was identified regarding the use of outreaching approaches to recruit older people with frailty and impairment in the design of digital health technologies. The most commonly used recruitment strategy in the current literature is recruitment through partners or by purposeful sampling. The risk of bias in selecting participants is higher when using these forms of recruitment than when using an outreaching approach. However, it is important to emphasize that the literature does not suggest how the outcome of studies is affected by the different strategies.

Another gap was identified in the description of the value of involvement and the outcome of the specific contribution of the participants. Thus, reflection on the use of different involvement methods in future work could help evolve the existing practices and enable more older people, who are not commonly included in development processes, to take part in future projects.

Finally, a series of different methods used to involve older people in the development of digital health technologies was identified. However, a gap was identified in the knowledge regarding the means to involve the older people in all parts of the design process, including the initial needs assessment phase. The literature does not imply which part of the development process involvement is most beneficial. However, only few studies included participants throughout the development process, and an overrepresentation of participants involved at the end of the design process and underrepresentation of participants involved in the first steps of the design process were identified.

## Appendix

Multimedia Appendix 1 Search strings.

Multimedia Appendix 2 Table S3. Recruitment strategies.

Multimedia Appendix 3 Table S4. Outcome of the study.

Multimedia Appendix 4 Table S5. Involvement methods.

## Abbreviations

AMD: age-related macular degeneration
EU: European Union
PRISMA-ScR: Preferred Reporting Items for Systematic Reviews and Meta-Analyses extension for Scoping Reviews
SMILE: Smart Inclusive Living Environments
WHO: World Health Organization
