# Supplementary material for: Involving Older People With Frailty or Impairment in the Design Process of Digital Health Technologies to Enable Aging in Place: Scoping Review
Source: JMIR Hum Factors. 2023 Jan 27;10:e37785. doi: 10.2196/37785 (PMC9919541; doi:10.2196/37785)
Supplement: Multimedia Appendix 1 [file humanfactors_v10i1e37785_app1.pdf]

PubMed Search String

(Telemedicine [MeSH] OR Digital Health OR eHealth OR Mobile Health OR mHealth OR Telehealth OR Smart Home OR Smart Health OR Gerontechnology OR welfare technology OR ambient assisted living OR assistive technology)
AND (Aged [MeSH] OR Elderly OR Ageing OR Older)
AND (Cognitive Dysfunction [MeSH] OR Cognitive Decline OR Cognitive Impairments OR Mental Deterioration OR Mild Cognitive Impairment OR Mild Neurocognitive Disorder OR Dementia OR Frail OR Disab* OR Disability OR Accessibility OR Motoric Dysfunction OR vulnerable)
AND (Co-creation OR Participatory Design OR User-Centered Design OR Co-creative OR user-Involvement OR co-design, co-production OR development OR innovation)
AND (Self-management OR Self-monitoring OR Empowerment OR Disempowerment OR Self-efficacy OR Coping OR ageing in place)

Embase

("Telemedicine" OR "Digital Health" OR "eHealth" OR "Mobile Health" OR "mHealth" OR "Telehealth" OR "Smart Home" OR "Smart Health" OR "Gerontotechnology" OR "Welfare technology" OR "Ambient Assisted Living" OR "Assistive Technology")
AND ("Aged" OR "Elderly" OR "Ageing" OR "Older")
AND ("Cognitive Dysfunction" OR "Cognitive Decline" OR "Cognitive Impairments" OR "Mental Deterioration" OR "Mild Cognitive Impairment" OR "Neurocognitive Disorder" OR "Dementia" OR "Frail" OR "Disabled" OR "Disab*" OR "Motoric Dysfunction" OR "Vulnerable")
AND ("Co-creation" OR "Participatory Design" OR "User-Centered Design" OR "Co-creative" OR "User-Involvement" OR "co-design" OR "co-production" OR "Development" OR "innovation") AND ("Self-management" OR "Self-monitoring" OR "Empowerment" OR "Disempowerment" OR "Self-efficacy" OR "Coping" OR "Ageing in place")
AND ("Digital Techn*" OR "Digital Health Care Techn*" OR "Digital Health Technology" OR "web-based")

IEEE

("Telemedicine" OR "Digital Health" OR "eHealth" OR "Mobile Health" OR "mHealth" OR "Telehealth" OR "Smart Home" OR "Smart Health" OR "Gerontechnology" OR "Welfare technology" OR "Ambient Assisted Living" OR "Assistive Technology") AND ("Aged" OR "Elderly" OR "Ageing" OR "Older")
AND ("Cognitive Dysfunction" OR "Cognitive Decline" OR "Cognitive Impairments" OR "Mental Deterioration" OR "Mild Cognitive Impairment" OR "Neurocognitive Disorder" OR "Dementia" OR "Frail" OR "Disabled" OR "Disab*" OR "Motoric Dysfunction" OR "Vulnerable")
AND ("Co-creation" OR "Participatory Design" OR "User-Centered Design" OR "Co-creative" OR "User-Involvement" OR "co-design" OR "co-production" OR "Development" OR "innovation")
AND ("Self-management" OR "Self-monitoring" OR "Empowerment" OR "Disempowerment" OR "Self-efficacy" OR "Coping" OR "Ageing in place")
AND ("Digital Techn*" OR "Digital Health Care Techn*" OR "Digital Health Technology" OR "web-based")

Scopus

(("Telemedicine" OR "Digital Health" OR "eHealth" OR "Mobile Health" OR "mHealth" OR "Telehealth" OR "Smart Home" OR "Smart Health" OR "Gerontechnology" OR "Welfare technology" OR "Ambient Assisted Living" OR "Assistive Technology")
AND ("Aged" OR "Elderly" OR "Ageing" OR "Older")
AND ("Cognitive Dysfunction" OR "Cognitive Decline" OR "Cognitive Impairments" OR "Mental Deterioration" OR "Mild Cognitive Impairment" OR "Neurocognitive Disorder" OR "Dementia" OR "Frail" OR "Disabled" OR "Disab*" OR "Motoric Dysfunction" OR "Vulnerable")
AND ("Co-creation" OR "Participatory Design" OR "User-Centered Design" OR "Co-creative" OR "User-Involvement" OR "co-design" OR "co-production" OR "Development" OR "innovation")
AND ("Self-management" OR "Self-monitoring" OR "Empowerment" OR "Disempowerment" OR "Self-efficacy" OR "Coping" OR "Ageing in place")
AND ("Digital Techn*" OR "Digital Health Care Techn*" OR "Digital Health Technology" OR "web-based")) AND (EXCLUDE (DOCTYPE , "re")) AND (LIMIT-TO (DOCTYPE , "ar") OR LIMIT-TO (DOCTYPE , "cp"))
