# Supplementary material for: Involving Older People With Frailty or Impairment in the Design Process of Digital Health Technologies to Enable Aging in Place: Scoping Review
Source: JMIR Hum Factors. 2023 Jan 27;10:e37785. doi: 10.2196/37785 (PMC9919541; doi:10.2196/37785)
Supplement: Multimedia Appendix 2 [file humanfactors_v10i1e37785_app2.pdf]

Table S3 Recruitment Strategies

**Article Information**	**Population Information**		**Recruitment Strategy**			
Author and year	*Description of population*	*Location/role of study population*	*Purposeful sampling*	*Outreach-ing*	*Invitation through partners*	*Strategy not described*
Albina et al 2018	60+ years, known physical or health condition	Home/out-pa-tients	x			
Alvarez et al 2020	65 years, older than 65 years old, no history of cognitive impair-ment, no visual impairment or adequately corrected by glasses or surgery, a cognitive assessment method (CAM) negative at study entry	Hospitalised			x	
Athilingam et al 2017	63 years (mean), heart Failure	Home/out-pa-tients				x
Bogza et al 2020	60-85+ years, mild cognitive impairment	Home/web-based				x
De Barros et al 2013	53-77 years, Parkinson disease	Home/out-pa-tients			x	
Du Preez et al 2019	65-83 years, with access to web-based services	Home/web-based			x	
Grossman et al 2018	55+ years, heart failure	Home/everyday life	x			
Govercin et al 2010	Three types of participants: (1) moderately to severely disabled participants with mild to severe risk of falling (average age 75 years). (2) slightly disabled older persons with low fall risk (aver-age age 68 years) and (3) healthy relatives of patients with severe risk of falling (average age 50 years).	Home			x	
Greenhalgh et al 2015	69-98 years, multi-morbidities	Home/out-pa-tients			x	
Hakobyan et al 2015	50+ years, AMD e.g., visual impairments, members of local AMD community groups-	Home/everyday life		x		
Hassan et al 2017	Four groups 1) people living with dementia and their carers >65 years .2) people living with memory problems or Mild Cognitive Impairment (MCI) and their carers >65 years. 3) people living with dementia aged <65 years referred to as young onset demen-tia. 4) people without known memory problems aged 50-70 years.	Home/everyday life		x		
Hoffman et al 2019	Advisory panel consisting of two older adults, two family caregiv-ers, two decision scientists, four informaticians, three geriatric psychiatrists, and three memory care specialists (16 people total)	Home/web-based			x	
Jacelon et al 2018	65+ years, living independently, and have one or more chronic conditions. Each participant was required to read and understand English	Home/everyday life			x	

Kerkhof et al 2019	72-86 years, community-dwelling care-dependent people with mild dementia, with and without a confirmed diagnosis. Inclusion criteria for the informal carers were caring for a person with possible dementia in an earlier stage. In the first and second sprint eight persons with dementia, eight informal carers and two formal carers participated in the usability tests	Home/everyday life	x	
Lehto et al 2013	60-94 years, elderly people (N=176) and experts (N=105). Of the elderly participants, 15 lived at home and the others lived in service houses or used the services at day centres. In addition, the focus group included 16 Swedish-speaking elderly people who participated in group activities at the day centres. The average age was 79.5 years.	Home		x
Macis et al 2018	65+ years, Chronic conditions two groups of older adults, randomly chosen from all the patients of a public health clinic in Italy, were involved in several test sessions. the inclusion criteria were limited to an age above 65 years, while exclusion criteria were the presence of physical impairment hampering the normal use of the system and declared cognitive deficits.<br><br>The evaluation of the TV-based version of the system involved a sample of 28 patients (14 males and 14 females) aged79±6years (67–93). Regarding education, three of them had a primary school certificate, 22 had a high-school diploma, and three had a university degree. The evaluation of the tablet-based version of the system involved a sample of 12 patients (4 males and 8 females) aged75±6years (65–83). Regarding education, three of them had a university degree, five had a high-school diploma, and the remaining four had a primary school certificate.	Home/everyday life		x
Oberschmidt et al 2020	64-88 years, Users and volounteers of a ‘BoodschappenPlusBus’ (=GroceryPlusBus, BPB) in the east of the Netherlands. The bus is an initiative of the Dutch National Foundation for the Elderly (NFE), which offers more than a hundred of these busses throughout the Netherlands. Participants can go on diverse trips, for example to a museum or the beach, but also to the supermarket or a mall. The trips are organised by volunteers who are mostly older adults as well.	Home/everyday life	x	
Pradhan et al 2020	65-76 years, six participants from a longitudinal study that deployed various IoT technologies, such as wearable activity monitors, motion sensors and door sensors, in the homes of retired older adults. Inclusion criteria specified that they must have had	Home/everyday life	x	

	these technologies in their homes for at least three months to ensure that they had experience with them beyond a novelty period. Participants were between 65 to 76 years in age, identified as females, and lived independently in their homes. All butP2had a college degree.			
Vanoh et al 2018	65+ years, older adults. the 73 subjects who took part in the study of acceptance consisted of 30 elderly people, nine health care experts, 32 caregivers, and two information technology (IT) experts chiefly working in web design. The selection of the elderly people for the study was based on the following factors: they must have at least secondary level of education (7 years or more), no dementia or any form of cognitive impairment, should have the basic know-how of using computer, should have computer/ipad/tablet at their place, and should have internet connection for accessing the website.	Home/web-based	x	
Wali et al 2020	60+ years, heart failure	Home/everyday life	x	
Wannheden & Revenäs 2020	73 years median, Parkinson disease	Home/everyday life		x
Willard et al 2018	65+ years, risk of cognitive decline	Home/web-based		x
