# Supplementary material for: Involving Older People With Frailty or Impairment in the Design Process of Digital Health Technologies to Enable Aging in Place: Scoping Review
Source: JMIR Hum Factors. 2023 Jan 27;10:e37785. doi: 10.2196/37785 (PMC9919541; doi:10.2196/37785)
Supplement: Multimedia Appendix 3 [file humanfactors_v10i1e37785_app3.pdf]

Table S4 Outcome of Study

Author and year	Study Aim	Olders Contribution to Study Outcome	Technology Developed
Albina et al 2018	Describe and understand needs, benefits, and barriers on assisted technology among elderlies for daily activities	not specified	Assistive Technology
Alvarez et al 2020	Develop a software for bedside use by hospitalised older adults designed to improve their access toa bundle of non-pharmacological interventions to prevent delirium.	Older assessed accessibility, this led to 7 features included in the app	A software application for touchscreen mobile devices
Athilingam et al 2017	Review the background and evidence in the development of a theory-based mobile app (HeartMap) and to improve self-care of patients with HF	User-involvement have changed both how to wear censors and the software	Interactive Mobile Health Application to Improve Self-care in Heart Failure
Bogza et al 2020	Develop a decision aid that support evidence-informed and value-based decision making for older adults with MCI	Identified and applied features that support values of participants and adjusted the level of detail of the web-based decision aid	Web-based decision aid platform to help decision-making among elderly's that are mild cognitively impaired
De Barros et al 2013	Develop four apps for smartphone, for self-management of Parkinson disease (PD) to 1) medication, 2) appointments, 3) my day (disease status/symptoms), 4) my data (personal and health information)	Develop app to monitor PD, with input from individuals with PD	Smartphone applications for self-management of PD
Du Preez et al 2019	Understand elders' perceptions of web-based services and technologies that could support aging in place and to inform the design of technology-enabled services	not specified	Web-based services (contribution to design of web-based services, product grounded theory)
Grossman et al 2018	Identify design requirements for an interface that assists patients with heart failure	Input from patients was used to identify design requirements for interface	Patient-Reported Outcomes (PRO) that uses visualizations to engage patients
Govercin et al 2010	To gather potential users' opinions on ICT-based visual and wearable fall prediction-, and prevention systems for home use for future system design and development of plant design	Participants consider fall prediction system to be as important as a fall detection system and prefer wearable on wrist. Inertial sensors preferred due to wide range of use over optical system	ICT-based visual and wearable fall prediction and fall prevention systems; wearables and optical system
Greenhalgh et al 2015	To understand the needs of older people with multi-morbidity and explore how technology suppliers, health, and social care providers can work with care recipients and carers to 'co-produce' development of new-, and adaption of existing technologies.	Reflect on participants values needs and experiences in relation to AUDDE (method)	Various assistive technologies
Hakobyan et al 2015	Aim through participatory, user-focused research to create a mobile assistive healthcare-related intervention for people with age-related macular degeneration (AMD) to promote independent living, enhanced well-being, and reduce risk of AMD progression based on dietary intake and associated recommendations	Focus groups with people with AMD (10), in-home observational studies (4), eight design meetings with people with AMD (4)	Mobile software application
Hassan et al 2017	Aim to involve members of the public in discussions about the acceptability and feasibility of different devices and research designs	Input in relation to acceptance and ease of use of different devices from possible end-	A software platform and device pool to support future connected health

	to inform the development of a device pool, software platform and written guidance to support future studies	users. e.g., resistance to water was seen as important across all groups	dementia research (testing various wearables (described in table 1)
Hoffman et al 2019	To develop a suite of decision aids to facilitate shared decision making, high-quality decisions, and person-centered care for older adults and their families	not specified	Decision aid for housing for older adults and spouses
Jacelon et al 2018	To develop ASSISTwell, an app for tablet designed for older individuals' self-management of symptoms of multiple chronic conditions	Based on input from end-users, a beta version of the user interface ASSISTwell was developed	Tablet application to support older adult's self-management of chronic conditions
Kerkhof et al 2019	To develop a person-centred selection tool 'Findmyapps' for to help people with mild dementia find supportive apps for self-management and meaningful activities	Used input from participants to design FindMyApps selection tool for tablet	FindMyApps selection tool for tablet - to help people with mild dementia find supportive apps for self-management
Lehto et al 2013	The aim of interactive CaringTV is to support the health and well-being of elderly living in own homes	End-user contributed to the development of the Interactive CaringTV platform	Interactive CaringTV platform to facilitate ageing-in-place
Macis et al 2018	usability assessment of the telemonitoring features of the HEREiAM platform, which supports heterogeneous information technology systems.	not specified	HEREiAM platform: Android-based system is designed to be accessible both via TV and portable devices
Oberschmidt et al 2020	Provides an overview of congruent and incongruent perspectives, interests or priorities from older adults, service and technology providers, researchers, and project management - how to reorganise existing services	Reflect on the methods used and the engagement of the participants and suggest 'interest mapping' and direct, iterative dialogue to aligning stakeholder interests.	Combining existing services in a new way
Pradhan et al 2020	Aim to understand how older adults with IoT experience would envision designing technologies for themselves	Find that the vision of the elder's shift depending on where in the design process they are etc.	Designing rehabilitation tools, creating new banking experiences or social virtual reality application
Vanoh et al 2018	Assessment of a web-based intervention for educating older people on strategies promoting healthy cognition with focus on technology acceptance	End-users assessed acceptance of WESIHAT 2.0 web-based tool	WESIHAT 2.0 - web-based tool (The components employed in WESIHAT 2.0 were a screening tool called TUA-WELLNESS)
Wali et al 2020	Aim to understand the self-care challenges, that older patients with HF and their informal care givers face daily.	Involvement led to 6 themes to consider when developing the mHealth App based on end-user needs	Conversion of 'SDDST' into a user-centred mHealth app
Wannheden & Revenäs 2020	Aim to explore how co-care could be operationalized in care of Parkinson disease, supported by eHealth.	Design of prototype based in co-design workshops and evaluated by end-users	eHealth prototype app for PwP designed for smartphone or tablet
Willard et al 2018	Aim to a) identify user requirements, b) modify an existing online platform, based on the needs of frail older adults, and in collaboration with a new supplier b) to test the modified prototype platform on the usability and the feasibility of frail older adults	not specified	Web-based platform: Online community care platform (OCC-platform) giving access to services
