# Supplementary material for: Involving Older People With Frailty or Impairment in the Design Process of Digital Health Technologies to Enable Aging in Place: Scoping Review
Source: JMIR Hum Factors. 2023 Jan 27;10:e37785. doi: 10.2196/37785 (PMC9919541; doi:10.2196/37785)
Supplement: Multimedia Appendix 4 [file humanfactors_v10i1e37785_app4.pdf]

Table S5 Involvement Methods

Article Information	Population	Involvement Methods in Design process				Other
Author and year	*Study Population Condition*	*Needs identification*	*Conceptualization*	*Prototyping*	*Evaluation of prototype*	*Other participants*
Athilingam et al 2017	Heart Failure	Interviews	Questionnaire	Feedback sessions	Usability testing	Cardiologists
De Barros et al 2013	Parkinson disease	Interviews	Scoping sessions	Focus groups	Usability testing	Informal care givers
Pradhan et al 2020	not specified	Interviews	Workshops, Take-home journal activity	Interviews		
Hoffman et al 2019	not specified	Focus groups Surveys		Testing and evaluation	Questionnaire	Family and caregivers (in initial phase)
Alvarez et al 2020	not specified		Two sprint sessions	Focus groups	Surveys	
Wannheden & Revenäs 2020	Parkinson Disease		Co-design workshops	Co-design workshops	Questionnaires after demonstration of prototype	Informal care givers
Lehto et al 2013	Poor health, functional ability, and forgetfulness	Interviews	Workshops		Observation	
Kerkhof et al 2019	Mild dementia			Tasks and interviews to identify usability problems	Usability testing (Interview-driven)	Development team + informal care givers
Grossman et al 2018	Heart failure	Interviews, Surveys			Usability testing	Health care providers
Willard et al 2018	Risk of cognitive decline	Observations, Interviews			Evaluation of platform	
Greenhalgh et al 2015	Multi-morbidities	Co-ethnography (cameras, diaries, and scrapbooks to collect data about participants lives), Observation		Co-design workshop		Service providers and tech providers
Hakobyan et al 2015	AMD e.g., visual impairments	Focus groups Observations		Design meetings		
Du Preez et al 2019	not specified	Interviews Workshops				
Albina et al 2018	Known physical or health condition	Surveys				
Macis et al 2018	Chronic conditions				Usability assessment, Interviews, Questionnaires	

Bogza et al 2020	Mild Cognitive Impairments		Questionnaire,<br>Interviews,<br>5-point rating scale	
Vanoh et al 2018	not specified		Assessment of ac-ceptance	Health care experts (9), caregivers (32), and it-experts (2)
Wali et al 2020	Heart Failure	Interviews		Care providers
Govercin et al 2010	Moderately and slightly disa-bled with severe/low risk of falling		Focus groups	Informal care givers
Hassan et al 2017	Dementia and cognitive im-pairments		Feedback sessions, workshops	
Jacelon et al 2018	Chronic conditions		Focus groups	
Oberschmidt et al 2020	not specified	Workshop		Facilitators helping with exercises
